# The Etiology of Pneumonia in HIV-infected Zambian Children: Findings From the Pneumonia Etiology Research for Child Health (PERCH) Study

**DOI:** 10.1097/INF.0000000000002649

**Published:** 2021-08-25

**Authors:** Phil Seidenberg, Lawrence Mwananyanda, James Chipeta, Geoffrey Kwenda, Justin M. Mulindwa, James Mwansa, Musaku Mwenechanya, Somwe Wa Somwe, Daniel R. Feikin, Meredith Haddix, Laura L. Hammitt, Melissa M. Higdon, David R. Murdoch, Christine Prosperi, Katherine L. O’Brien, Maria Deloria Knoll, Donald M. Thea

**Affiliations:** From the * Department of Global Health, Boston University School of Public Health, Boston, Massachusetts; † Department of Emergency Medicine, University of New Mexico, Albuquerque, New Mexico; ‡ Right To Care-Zambia, Lusaka, Zambia; § Department of Paediatrics and Child Health, University of Zambia School of Medicine, Lusaka, Zambia; ¶ Department of Paediatrics, University Teaching Hospital, Lusaka, Zambia; ‖ Department of Biomedical Sciences, School of Health Sciences, University of Zambia, Lusaka, Zambia; ** Department of Pathology and Microbiology, University Teaching Hospital, Lusaka, Zambia; †† Department of Microbiology, Lusaka Apex Medical University, Lusaka, Zambia; ‡‡ Department of International Health, International Vaccine Access Center, Johns Hopkins Bloomberg School of Public Health, Baltimore, Maryland; §§ Department of Pathology, University of Otago, Christchurch, New Zealand; ¶¶ Microbiology Unit, Canterbury Health Laboratories, Christchurch, New Zealand.

**Keywords:** Zambia, pneumonia, etiology, childhood, PERCH, HIV

## Abstract

Despite recent declines in new pediatric HIV infections and childhood HIV-related deaths, pneumonia remains the leading cause of death in HIV-infected children under 5. We describe the patient population, etiology and outcomes of childhood pneumonia in Zambian HIV-infected children.

**Methods:**

As one of the 9 sites for the Pneumonia Etiology Research for Child Health study, we enrolled children 1–59 months of age presenting to University Teaching Hospital in Lusaka, Zambia, with World Health Organization–defined severe and very severe pneumonia. Controls frequency-matched on age group and HIV infection status were enrolled from the Lusaka Pediatric HIV Clinics as well as from the surrounding communities. Clinical assessments, chest radiographs (CXR; cases) and microbiologic samples (nasopharyngeal/oropharyngeal swabs, blood, urine, induced sputum) were obtained under highly standardized procedures. Etiology was estimated using Bayesian methods and accounted for imperfect sensitivity and specificity of measurements.

**Results:**

Of the 617 cases and 686 controls enrolled in Zambia over a 24-month period, 103 cases (16.7%) and 85 controls (12.4%) were HIV infected and included in this analysis. Among the HIV-infected cases, 75% were <1 year of age, 35% received prophylactic trimethoprim-sulfamethoxazole, 13.6% received antiretroviral therapy and 36.9% of caregivers reported knowing their children’s HIV status at time of enrollment. A total of 35% of cases had very severe pneumonia and 56.3% had infiltrates on CXR. Bacterial pathogens [50.6%, credible interval (CrI): 32.8–67.2], *Pneumocystis jirovecii* (24.9%, CrI: 15.5–36.2) and *Mycobacterium tuberculosis* (4.5%, CrI: 1.7–12.1) accounted for over 75% of the etiologic fraction among CXR-positive cases. *Streptococcus pneumoniae* (19.8%, CrI: 8.6–36.2) was the most common bacterial pathogen, followed by *Staphylococcus aureus* (12.7%, CrI: 0.0–25.9). Outcomes were poor, with 41 cases (39.8%) dying in hospital.

**Conclusions:**

HIV-infected children in Zambia with severe and very severe pneumonia have poor outcomes, with continued limited access to care, and the predominant etiologies are bacterial pathogens, *P. jirovecii* and *M. tuberculosis.*

## Abstract 2

Supplemental Digital Content is available in the text.

In the past 10 years, significant declines in new pediatric HIV infections and childhood HIV-related deaths have occurred, largely due to widespread access to highly efficacious prevention of mother to child transmission (PMTCT) regimens, as well as highly active antiretroviral therapy for children.^1^ Despite these efforts, in 2016, there were an estimated 2.1 million children (<15 years) living with HIV and 160,000 new infections, the overwhelming majority (90%) living in Sub-Saharan Africa where the epidemic is most prominent.^1^ The leading cause of morbidity and mortality in these children is pneumonia.^2–4^ HIV-infected children are more likely to be hospitalized from pneumonia than any other illness,^5,6^ are more likely to fail antimicrobial treatment^7,8^ and are more likely to suffer worse outcomes (including death) from pneumonia than HIV-uninfected children.^7,9,10^ In addition to common causative pathogens found in childhood community-acquired pneumonia [CAP; *Streptococcus pneumoniae*, *Haemophilus influenzae*, respiratory syncytial virus (RSV)], HIV-infected children were also found to be susceptible to opportunistic pathogens such as *Pneumocystis jirovecii* (Pj), cytomegalovirus (CMV) and *Mycobacterium tuberculosis* (MTB).^2,4,11,12^

Because of these factors, the 2014 World Health Organization (WHO) recommendations for health facility treatment were updated for empiric antimicrobial treatment of CAP in HIV-infected children.^2,13^ Unfortunately, the majority of studies used to support these recommendations were conducted before widely available highly active antiretroviral therapy in children, the standard use of trimethoprim-sulfamethoxazole (CTM) prophylaxis for HIV-infected and HIV-exposed children,^14^ and the routine *H. influenzae* type b (Hib) and pneumococcal conjugate (PCV) vaccination programs in HIV-epidemic areas.

The aim of this analysis is to describe the etiology and outcomes of childhood pneumonia in Zambian HIV-infected children included in the previously described Pneumonia Etiology Research for Child Health (PERCH) study.^15^ A similar PERCH analysis of Zambian HIV-uninfected children is also presented in this supplement.^16^

## MATERIALS AND METHODS

### Location

The Zambia PERCH study site was located at the University Teaching Hospital (UTH) in the densely populated capital, Lusaka (population 1.7 million^17^). While Zambia is considered a lower-middle-income country (per capita income $4300), at the time of the study (November 2011 to October 2013), approximately 74% of the country’s population was living in extreme poverty (<$2/d).^18^ UTH provides free health services to the most impoverished in Lusaka. As the primary academic and tertiary healthcare facility and the main country-wide referral center, UTH has a 425-bed in-patient pediatric ward with dedicated Malnutrition and Intensive Care Units. Access to mechanical ventilation was limited and rarely used. Obtaining radiographs required taking children to the Radiology Department at some distance from the pediatric wards and at the caregivers’ expense, therefore, were not routinely performed (for nonstudy patients). Oxygen, however, was routinely available. The majority of children presenting for pneumonia care at UTH are referred from outlying Lusaka clinics after receiving 1 dose of antibiotics.

Hib conjugate vaccine was introduced in 2004 with 81% estimated 3-dose coverage before the study.^19^ PCV10 was only universally introduced in July 2013 during the final 3 months of study enrollment.

In 2013, HIV prevalence in Lusaka among women of childbearing age was 19.4%.^20^ Antenatal PMTCT care was nearly universal (91%) in Zambia, leading to a decline in vertical HIV transmission from 24% in 2009 to 12% by 2012.^21^ By 2013, an estimated 54% of the 150,000 Zambian HIV-infected children were accessing antiretroviral therapy (ART).^22^ While pediatric HIV seroprevalence rates at UTH were unavailable at the time of PERCH, a 2007 study found 29.2% HIV antibody positive rate among over 11,500 children tested in the pediatric ward.^23^

At the time of PERCH, the standard of care for HIV-infected children <2 years was to initiate ART regardless of CD4 count or clinical staging.^24^ For 2- to 5-year-old children, ART was initiated if the CD4 count was ≤750 cells/mm^3^, CD4 percentage <25% or clinical concern for advanced disease based on WHO staging.^24^ The most common first-line ART regimen consisted of lopinavir/ritonavir plus 2 nucleoside reverse transcriptase inhibitors. CTM prophylaxis beginning at 4–6 weeks of age was universally recommended and available for all HIV-infected and exposed children.

### Participants

PERCH methods have been published elsewhere.^15,25^ Unless otherwise noted, cases for this analysis were HIV-infected hospitalized children between the ages of 1 and 59 months living in the Lusaka catchment area presenting with signs and symptoms of WHO-defined severe or very severe pneumonia (2005 definition), including cough and/or difficulty in breathing, plus danger signs (central cyanosis, difficulty breast-feeding/drinking, vomiting everything, multiple or prolonged convulsions, lethargy/unconsciousness or head nodding) defined as “very severe pneumonia,” or lower chest wall indrawing in the absence of danger signs defined as “severe pneumonia.”^26^ Cases were enrolled on weekdays from 0730 to 1800 hours due to weekend constraints for sample processing and limited weekend and nighttime staffing. Nighttime admissions were eligible for study enrollment the following morning.

HIV-infected controls, recruited from Pediatric HIV clinics in the Lusaka area without evidence of pneumonia, were age-frequency matched to HIV-infected cases using 4 strata: 1–5, 6–11, 12–23 and 24–59 months. A few HIV-infected controls were (incidentally) enrolled during routine community control recruitment.^16^

### Clinical Procedures

Cases were examined at admission and 24 and 48 hours postadmission. Cases that survived to discharge were seen 30 days after discharge to ascertain vital status. Chest radiographs (CXRs) were performed at admission and classified as normal, consolidation, other infiltrate, consolidation and other infiltrate or uninterpretable based on WHO methods.^27,28^ Clinical assessments of controls were completed at the time of enrollment.

### Specimen Collection and Laboratory Methods

Specimen collection and laboratory methods were highly standardized across study sites.^29–33^ From all participants, we collected nasopharyngeal/oropharyngeal (NP/OP) swabs for polymerase chain reaction (PCR) for respiratory pathogens using a 33-pathogen multiplex quantitative PCR (FTD Resp-33; Fast-track Diagnostics, Sliema, Malta) and culture (plus serotyping) for pneumococcus, blood for pneumococcal PCR and serum for antibiotic activity testing.^31^ From cases, we also collected blood for bacterial culture and induced sputum for MTB culture. For four pathogens with similar prevalence in cases and controls, positivity was defined using quantitative PCR density thresholds; including *S. pneumoniae* (≥2.2 log10 copies/mL) from whole blood^34^ and *S. pneumoniae* (≥6.9 log10 copies/mL),^35^ *H. influenzae* (≥5.9 log10 copies/mL),^36^ CMV (≥4.9 log10 copies/mL) and Pj (≥4 log10 copies/mL),^36^ NP/OP (CMV threshold analysis available from authors). Maternal HIV status was obtained from the infant perinatal card or if the mother indicated she was HIV-infected. The child’s blood was tested by PCR if <18 months or HIV antibody if ≥18 months as per Zambian guidelines.

### Statistical Analysis

Odds ratios (OR) and 95% confidence intervals (CI) of pathogens detected on NP/OP PCR in cases compared with controls were calculated using logistic regression adjusted for age in months and presence of all other pathogens detected on NP/OP PCR to account for associations between pathogens. Logistic regression adjusted for age in months was used to compare clinical characteristics by case–control status and, among cases, by vital status. Results were stratified by HIV infection and exposure status.

The percent of pneumonia due to each pathogen was estimated using the PERCH Integrated Analysis (PIA) method, which is described in detail elsewhere (see reference 39, Appendix Section III B).^37–39^ In brief, the PIA is a Bayesian nested partially latent class analysis that integrates the results for each case from blood culture, NP/OP PCR, whole-blood PCR for pneumococcus and induced sputum culture for MTB. The PIA also integrates test results from controls to account for imperfect test specificity of NP/OP PCR and whole-blood PCR. Blood culture results (excluding contaminants) and MTB results were assumed to be 100% specific (ie, the etiology for a case was attributed 100% to the pathogen that was detected in their blood by culture). The model assumes that each child's pneumonia was caused by a single pathogen.

The PIA accounts for imperfect sensitivity of each test/pathogen measurement by using a priori estimates of their sensitivity (ie, estimates regarding the plausibility range of sensitivity which varied by laboratory test method and pathogen) (Supplemental Digital Content 1, http://links.lww.com/INF/D818). Sensitivity of blood culture was reduced if blood volume was low (<1.5 mL) or if antibiotics were administered before specimen collection. Sensitivity of NP/OP PCR for *S. pneumoniae* and *H. influenzae* was reduced if antibiotics were administered before specimen collection.

As a Bayesian analysis, both the list of pathogens and their starting “prior” etiologic fraction values were specified a priori, which favored no pathogen over another (ie, “uniform”). The pathogens selected for inclusion in the analysis included any noncontaminant bacteria detected by culture in blood at any of the 9 PERCH sites, regardless of whether it was observed at the Zambia site specifically, MTB, and all of the multiplex quantitative PCR pathogens except those considered invalid because of poor assay specificity (*Klebsiella pneumoniae*^40^ and *Moraxella catarrhalis*). A category called “Pathogens Not Otherwise Specified” was also included to estimate the fraction of pneumonia caused by pathogens not tested for or not observed. A child negative for all pathogens would still be assigned an etiology, which would be either one of the explicitly estimated pathogens (implying a “false negative,” accounting for imperfect sensitivity of certain measurements) or “Pathogens Not Otherwise Specified.”

All analyses were adjusted for age (<1 vs. ≥1 year) to account for differences in pathogen prevalences by this factor; analyses stratified by pneumonia severity could not adjust for age due to small sample size. For results stratified by case clinical data (eg, to CXR+, very severe, etc), the test results from all controls were used. However, for analyses stratified by age, only data from controls representative of that age group were used.

The PIA estimated both the individual and population-level etiology probability distributions, each summing to 100% across pathogens where each pathogen has a probability ranging from 0% to 100%. The population-level etiologic fraction estimate for each pathogen was approximately the average of the individual case probabilities and was provided with a 95% credible interval (95% CrI), the Bayesian analog of the confidence interval. Statistical analyses were conducted using SAS 9.3 (SAS Institute, Cary, NC), R Statistical Software 3.3.1 (The R Development Core Team, Vienna, Austria), Bayesian inference software JAGS 4.2.0 (http://mcmc-jags.sourceforge.net/) and BAKER, the R package used to perform the PIA (https://github.com/zhenkewu/baker).

### Ethical Considerations

The study protocol was approved by the Institutional Review Boards at Boston University, the Johns Hopkins Bloomberg School of Public Health in the United States, and by the ERES Converge Ethical Review Committee in Zambia. Parents or guardians of participants provided written informed consent.

## RESULTS

### Study Participants

**FIGURE 1.**
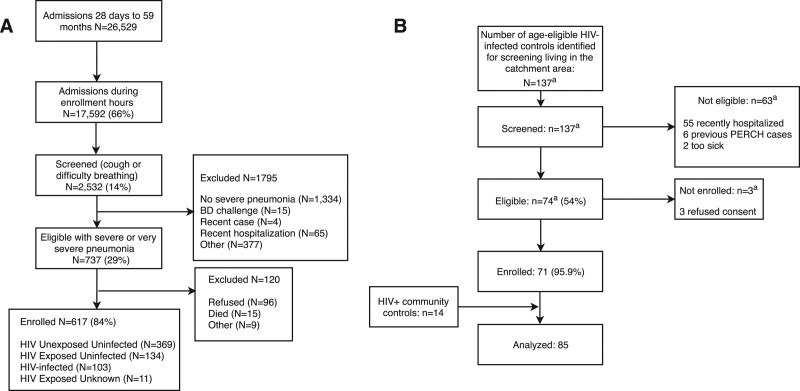
Case (A) and control (B) enrollment flow diagram. HIV-infected community controls reflect controls enrolled into the PERCH study from the community who were HIV-infected. For analysis purposes, the HIV-infected community controls are combined with the controls enrolled from the HIV clinic to form the full set of HIV-infected controls. ^a^Numbers estimated for 8 months. BD indicates bronchodilator.

Of the 617 children enrolled with severe and very severe pneumonia (Fig. 1), 369 (59.8%) were HIV-unexposed and uninfected (HUU), 134 (21.7%) were HIV-exposed and uninfected (HEU) and 103 (16.7%) were HIV-infected. Of 686 controls, 85 (12.4%) were HIV-infected [71 recruited from Pediatric HIV Clinics and 14 (16.5%) incidentally recruited during community enrollment] and 46 (50.6%) had upper respiratory tract illness symptoms. Unless specified, analyses are limited to HIV-infected participants. HIV-infected cases were young (75% <1 year old) and younger than controls (54.1%, *P* = 0.003) despite efforts to age-frequency match (Table 1).

**TABLE 1.** Demographic and Clinical Characteristics of HIV-infected Cases and Controls

	All Cases	CXR+ Cases	Controls
All	103	58	85
Age			
Median age in months (IQR)	6 (3, 12)	6.5 (3, 13)	11 (5, 24)
28 d–5 mo	51 (49.5)	27 (46.6)	24 (28.2)
6–11 mo	26 (25.2)	14 (24.1)	22 (25.9)
12–23 mo	13 (12.6)	10 (17.2)	14 (16.5)
24–59 mo	13 (12.6)	7 (12.1)	25 (29.4)
Sex			
Female	55 (53.4)	29 (50.0)	39 (45.9)
Season of enrollment			
Dry (June–October)	60 (58.3)	34 (58.6)	37 (43.5)
Rainy (November–May)	43 (41.7)	24 (41.4)	48 (56.5)
Respiratory tract illness (controls only)^a^	-	-	43 (50.6)
Pentavalent (DTP-Hib-HepB) fully vaccinated for age^b^			
<1-yr old	54 (73.0)	29 (74.4)	34 (79.1)
≥1-yr old	15 (83.3)	8 (72.7)	21 (84.0)
Total	69 (75.0)	37 (74.0)	55 (80.9)
Premature^c^	6 (5.8)	5 (8.6)	10 (12.1)
HIV characteristics^d^			
Child not reported to be HIV-infected	65 (63.1)	36 (62.1)	9 (10.6)
Not receiving prophylactic trimethoprim-sulfamethoxazole	67 (65.0)	37 (63.8)	25 (29.4)
Not on HAART	89 (86.4)	48 (82.8)	50 (58.8)
Median weeks on HAART (IQR)	17.7 (5.3–49.3)	28.4 (5.4–49.3)	22.5 (9.6–58.1)
Attended HAART Clinic in last 3 mo	19 (18.5)	13 (22.4)	40 (47.1)
Had CD4 count measured in last 3 mo	7 (6.8)	4 (6.9)	31 (36.5)
Moderate or severe malnutrition (weight-for-age)^e^	65 (63.1)	38 (65.5)	31 (36.5)
Antibiotic pretreatment before specimen collection^f^	90 (88.2)	49 (86.0)	8 (9.5)
Serum antibiotic activity	36 (36.4)	19 (33.9)	8 (10.7)
Very severe pneumonia^g^	36 (35.0)	17 (29.3)	-
CXR available	84 (81.6)	58 (100)	-
CXR result			
Any consolidation	50 (59.5)	50 (86.2)	-
Other infiltrate only	8 (9.5)	8 (13.8)	-
Normal	9 (10.7)	-	-
Uninterpretable	17 (20.2)	-	-
CRP ≥ 40 mg/L	46 (48.4)	30 (55.6)	-
Median CRP (mg/L) (IQR)	31.0 (5.9–110.4)	56.2 (8.8–171.6)	-
Severe anemia^h^	29 (29.0)	14 (24.1)	-
Leukocytosis^i^	49 (49.0)	33 (56.9)	-
Hypoxia^j^	61 (59.8)	36 (63.2)	-
Elevated temperature (≥38°C)	63 (61.2)	34 (58.6)	-
Tachycardia	73 (71.6)	40 (70.2)	-
Wheeze on auscultation	6 (5.8)	6 (10.3)	-
Lethargy^k^	17 (16.5)	6 (10.3)	-
Median duration of illness in days (IQR)^l^	3 (2, 7)	4 (2, 7)	-
Duration of illness^l^			
0–2 d	30 (29.1)	15 (25.9)	-
3–5 d	35 (34.0)	21 (36.2)	-
>5 d	38 (36.9)	22 (37.9)	-
Median duration of hospitalization in days (IQR)	6 (3, 13)	7 (4, 13)	-
Duration of hospitalization			
0–2 d	21 (20.6)	6 (10.5)	-
3–5 d	24 (23.5)	17 (29.8)	-
>5 d	57 (55.9)	34 (59.6)	-
Died in hospital	41 (39.8)	16 (27.6)	-
Died postdischarge, within 30 d of admission	2 (6.5)	1 (5.6)	-
Missing 30-d vital status^m^	31 (50.0)	24 (57.1)	-

^a^Respiratory tract illness was defined as the presence of cough or runny nose, or if a child had (1) at least 1 of ear discharge, wheezing or difficulty breathing and (2) either a measured temperature of >38.0°C within the previous 48 h or a history of sore throat.

^b^Pentavalent vaccine (DTP-Hib-HepB) used in Zambia. For children <1 yr, defined as received at least 1 dose and up-to-date for age based on the child’s age at enrollment, doses received and country schedule (allowing 4-wk window each for dose). For children >1 yr, defined as ≥3 doses.

^c^Prematurity defined as <37 wk gestational age or maternal report of premature.

^d^HIV characteristics that were missing were assumed to be negative.

^e^Moderate or severe malnutrition defined as less than −2 SD weight-for-age Z scores.

^f^Defined as serum bioassay positive (cases and controls), antibiotics administered at the referral facility or antibiotic administration before whole-blood specimen collection at the study facility (cases only).

^g^Very severe pneumonia defined as cough or difficulty breathing, and at least one of the following: central cyanosis, difficulty breast-feeding/drinking, vomiting everything, convulsions, lethargy, unconsciousness or head nodding.

^h^Severe anemia defined as hemoglobin < 7.5 g/dL.

^i^Leukocytosis count defined as >15 × 10^9^ cells/L for children 1–11 mo and >13 × 10^9^ cells/L for children 12–59 mo.

^j^Hypoxemia defined as oxygen saturation <90% or on supplemental oxygen if a room air oxygen saturation reading was not available.

^k^Lethargic or unresponsive (responds to voice or pain, unresponsive or pharmacologically sedated).

^l^Duration of illness defined as duration (in days) of cough, wheeze, fever or difficulty breathing, whichever is longest.

^m^Restricted to those children discharged alive.

CRP indicates C-reactive protein; CXR, chest radiograph; DTP, diphtheria-tetanus-pertussis vaccine; HAART, highly active antiretroviral therapy; HIV, human immunodeficiency virus; IQR, interquartile range.

Only 36.9% (27.3% for children <1 year) of case caregivers reported knowing that their child was HIV-infected (Table 1). Pediatric HIV clinic attendance in the past 3 months was low in cases (18.5%) compared with controls (47.1%; *P* < 0.0001), as was being on ART (13.6% vs. 41.2%) and receiving CTM prophylaxis (35.0% vs. 70.6%; both *P* < 0.0001); only 2.6% of cases <1 year were receiving ART. Restricting to those children whose caregivers knew their children’s HIV status, these characteristics were similar between cases and controls (Supplemental Digital Content 2, http://links.lww.com/INF/D819). CD4 count data were largely unavailable and viral load testing was not routine during PERCH.

Both cases (75%) and controls (81%) were up-to-date for pentavalent vaccine. More cases (63.1%) than controls (36.5%; *P* = 0.0002) were moderately-to-severely malnourished (Table 1). Because of common referrals to UTH, almost all cases (92.2%) received antibiotics before enrolment and specimen collection.

### Case Characteristics and Outcomes

Of all 103 cases, 35% had pneumonia danger signs (ie, very severe) with a median preceding duration of illness of 3 days and 39.8% died in hospital (41.5% within 48 hours). Most were hypoxic (59.8%), febrile (61.2%) and tachycardic (71.6%) (Table 1). Few (5.8%) had auscultatory wheeze. Of 84 (81.6%) cases with available CXRs, 50 (59.5%) showed evidence of consolidation or other infiltrate. Severe anemia, leukocytosis and elevated C-reactive protein were also common (29%–49%; Table 1). Only 19 children who died had an interpretable CXR, of which 84% showed evidence of consolidation or other infiltrate (Table 2). Factors significantly associated with in-hospital mortality after adjusting for age were infancy [adjusted odds ratio (aOR): 2.8, 95% CI: 1.0–7.7], grunting (aOR: 2.7, 95% CI: 1.1–6.6), lethargy (aOR: 3.3, 95% CI: 1.1–9.9) and pneumonia severity (aOR: 2.4, 95% CI: 1.0–5.6); hypoxia was very common among fatal cases but nonsignificant (73.2% vs. 50.8%; *P* = 0.08; Table 2). In-hospital mortality was significantly higher among HIV-infected children compared with HIV-uninfected exposed (21.6%) and HIV-unexposed (11.1%) (*P* < 0.0001) (Supplemental Digital Content 3, http://links.lww.com/INF/D820).

**TABLE 2.** Clinical and Laboratory Factors Associated With In-Hospital Mortality Among 103 HIV-infected <5-yr-Old Zambian Children With Severe or Very Severe Pneumonia on Admission

Characteristic	Fatal Cases	Nonfatal Cases	aOR^a^	*P*
	41	62		
Female	22 (53.7)	33 (53.2)	1.1 (0.5–2.4)	0.87
Very severe pneumonia^b^	20 (48.8)	16 (25.8)	2.4 (1.0–5.6)	**0.048**
Age < 1 yr	35 (85.4)	42 (67.7)	2.8 (1.0–7.7)	**0.049**
Weight-for-age < −2 SD	26 (63.4)	39 (62.9)	1.4 (0.6–3.3)	0.49
Weight-for-height < −2 SD	13 (34.2)	19 (31.7)	1.4 (0.6–3.3)	0.49
Premature^c^	1 (2.9)	3 (7.1)	0.4 (0.0–3.9)	0.41
Duration of illness^d^				
0–2 d	13 (31.7)	17 (27.4)	Ref	0.10
3–5 d	9 (22.0)	26 (41.9)	0.5 (0.2–1.6)	
>5 d	19 (46.3)	19 (30.6)	1.7 (0.6–4.6)	
Duration in hospital				
≤2 d	19 (46.3)	2 (3.3)	14.0 (2.6–76.1)	**0.0001**
3–5 d	9 (22.0)	15 (24.6)	Ref	
>5 d	13 (31.7)	44 (72.1)	0.5 (0.2–1.3)	
CXR positive^e^				
Consolidation or other infiltrate	16 (84.2)	42 (87.5)	0.8 (0.2–3.7)	0.76
Normal	3 (15.8)	6 (12.5)	Ref	
Runny nose	8 (19.5)	23 (37.1)	0.3 (0.1–0.9)	**0.026**
Hypoxia^f^	30 (73.2)	31 (50.8)	2.2 (0.9–5.3)	0.082
Lethargy	11 (26.8)	6 (9.7)	3.3 (1.1–9.9)	**0.034**
Deep breathing	5 (12.2)	3 (4.8)	2.3 (0.5–10.6)	0.27
Observed cough	26 (63.4)	44 (71.0)	0.8 (0.3–1.8)	0.52
Observed grunting	18 (43.9)	14 (22.6)	2.7 (1.1–6.6)	**0.026**
Severe anemia^g^	14 (35.9)	15 (24.6)	2.2 (0.9–5.6)	0.10
Leukocytosis^h^	18 (46.2)	31 (50.8)	0.8 (0.3–1.8)	0.59

^a^Odds ratios adjusted for age in months (aOR).

^b^Very severe pneumonia defined as cough and/or difficulty in breathing, plus danger signs (central cyanosis, difficulty breast-feeding/drinking, vomiting everything, multiple or prolonged convulsions, lethargy/unconsciousness or head nodding). Severe pneumonia defined as lower chest wall indrawing in the absence of danger signs.

^c^Prematurity defined as <37 wk gestational age or maternal report of premature.

^d^Duration of illness defined as duration (in days) of cough, wheeze, fever or difficulty breathing, whichever is longest.

^e^Restricted to those with an interpretable CXR (N = 19 for fatal cases and N = 48 for nonfatal cases). CXR obtained at admission.

^f^Hypoxemia defined as oxygen saturation <90% or on supplemental oxygen if a room air oxygen saturation reading was not available.

^g^Severe anemia defined as hemoglobin <7.5 g/dL.

^h^Leukocytosis count defined as >15 × 10^9^cells/L for children 1–11 mo and >13 × 10^9^ cells/L for children 12–59 mo.

aOR indicates adjusted odds ratio; CXR, chest radiograph.

Bold indicates *P* < 0.05.

### Specimen Microbiology and PCR Results

Blood cultures were positive in 8 (7.8%) cases, 6 (10.5%) of those with abnormal CXR findings and 3 (7.3%) of in-hospital fatal cases (Supplemental Digital Content 4, http://links.lww.com/INF/D821). Five (62.5%) were *S. pneumoniae*, all PCV10-serotype, and 1 each were *H. influenzae* (nontype b), *Salmonella* species and *K. pneumoniae*. Of 76 (73.8%) cases with induced sputum specimens, only 1 (1.3%) showed MTB, a 12-month-old child with abnormal CXR findings who died. Blood culture positivity was more common among HIV-infected children compared with HIV-uninfected children, driven by differences in *S. pneumoniae* detection (Supplemental Digital Content 5, http://links.lww.com/INF/D822).

Over 90% of cases with radiographic pneumonia had 3 or more organisms detected on NP/OP PCR (Supplemental Digital Content 6, http://links.lww.com/INF/D823). After applying the NP/OP PCR density thresholds, 71.7% of cases had 3 or more organisms detected, with only 2 cases being negative for all. Pathogens associated with CXR+ cases compared with controls after adjusting for age and codetection of other pathogens included: Pj (30.2% in cases, aOR = 5.3), *H. influenzae* nontype b (≥5.9 log10 copies/mL) (28.3%, aOR 4.4), *Staphylococcus aureus* (20.8%, aOR = 4.2), RSV (13.2%, aOR = 10.2) and adenovirus (9.4%, aOR = 20.3) (Supplemental Digital Content 7, http://links.lww.com/INF/D824). One CXR+ case tested positive for malaria. Differences by HIV status in NP/OP PCR prevalence and association with case status were observed for certain pathogens (Supplemental Digital Content 8, http://links.lww.com/INF/D825).

### Etiologic Distribution

**FIGURE 2.**
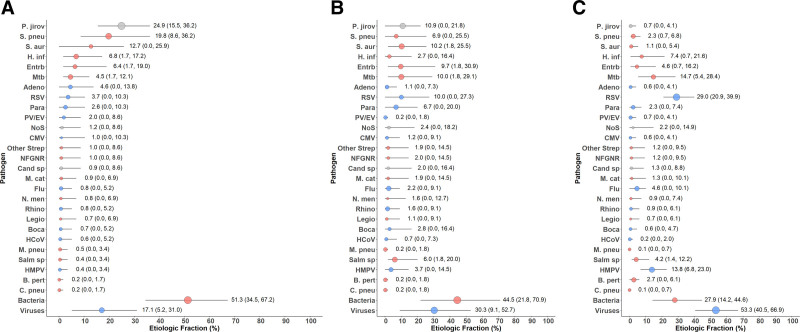
Integrated etiology results for (A) HIV-infected, (B) HIV-exposed and (C) HIV-unexposed CXR+ cases. Sample size: CXR+/HIV-infected: N = 58; CXR+/HIV-exposed: N = 55; CXR+/HIV-unexposed: N = 151. Line represents the 95% credible interval. The size of the symbol is scaled on the basis of the ratio of the estimated etiologic fraction to its standard error. Of 2 identical etiologic fraction estimates, the estimate associated with a larger symbol is more informed by the data than the priors. Bacterial summary excludes Mtb. Pathogens that were estimated at the subspecies level but grouped to the species level for display include Parainfluenza virus type 1, 2, 3 and 4; *S. pneumoniae* PCV10 and *S. pneumoniae* non-PCV10 types; *H. influenzae* type b and *H. influenzae* non b; and influenza A, B and C. Etiologic fraction estimates, including subspecies and serotype disaggregation (eg, PCV10 type and non-PCV10 type), are given in Supplemental Digital Content 9, http://links.lww.com/INF/D826. Adeno indicates adenovirus; B. pert, *Bordetella pertussis*; Boca, human bocavirus; C. pneu, *Chlamydophila pneumoniae*; Cand sp, *Candida* species; CMV, cytomegalovirus; Entrb, enterobacteriaceae; Flu, influenza virus A, B and C; H. inf, *Haemophilus influenzae*; HCoV, Coronavirus; HMPV, human metapneumovirus A/B; Legio, *Legionella* species; M. cat, *Moraxella catarrhalis*; M. pneu, *Mycoplasma pneumoniae*; Mtb, *Mycobacterium tuberculosis*; NFGNR, nonfermentative Gram-negative rods; N. men, *Neisseria meningitidis*; NoS, not otherwise specified (ie, pathogens not tested for); P. jirov, *P. jirovecii*; Para, parainfluenza virus types 1, 2, 3 and 4; PV/EV, parechovirus/enterovirus; Rhino, human rhinovirus; RSV, respiratory syncytial virus A/B; S. aur, *Staphylococcus aureus*; S. pneu, *Streptococcus pneumoniae*; Salm sp, *Salmonella* species. Other Strep includes *Streptococcus pyogenes* and *Enterococcus faecium*. NFGNR includes *Acinetobacter* species and *Pseudomonas* species. Enterobacteriaceae includes *Escherichia coli*, *Enterobacter* species and *Klebsiella* species, excluding mixed Gram-negative rods.

Bacterial pathogens (50.6%, 95% CrI 32.8–67.2) and Pj (24.9%, 95% CrI: 15.5–36.2) accounted for over 70% of the etiologic fraction of CXR+ pneumonia, with viruses contributing only 17.1% (95% CrI: 5.2–31.0) (Figure 2 and Supplemental Digital Content 9, http://links.lww.com/INF/D826). *S. pneumoniae* (19.8%, 95% CrI: 8.6–36.2), *S. aureus* (12.7%, 95% CrI: 0–25.9) and Pj were the most common pathogens, almost twice as common as the next most frequent, *H. influenzae* (6.8%, CrI: 1.7–17.2).

Six of the top 10 pathogens were potentially treatable: Pj, *S. pneumoniae*, *S. aureus, H. influenzae*, Enterobacteriaceae (6.4%, 95% CrI: 1.7–19.0) and MTB (4.5%, 95% CrI: 1.7–12.1), cumulatively accounting for 75.2% (95% CrI: 56.9–89.7) of etiology.

Analyses stratified by age and severity were limited by sample size (Supplemental Digital Content 10, http://links.lww.com/INF/D827, and Supplemental Digital Content 11, http://links.lww.com/INF/D828). Notable significant differences included Pj being uncommon in children ≥1 year (0.2% vs. <1 year, 35.2%), *H. influenzae* non-b being uncommon in <1 year (0.7% vs. ≥1 year, 18.0%), *S. pneumoniae* being more common among severe (22.8%) versus very severe (4.3%) and RSV being uncommon in very severe (0.4% vs. severe 7.8%); Pj was more common among very severe cases (37.0% vs. severe 20.0%) but not significant (mean difference 17.0%, 95% CrI: −0.9 to 40.3).

HIV-infected CXR+ cases had a greater proportion etiology attributed to bacteria than HUU cases (27.2, 95% CrI: 13.5–43.9), primarily due to *S. pneumoniae* and *S. aureus*, and Pj was virtually nonexistent among HUU (0.7, 95% CrI: 0.0–4.1; Figure 2). The fraction attributed to RSV was small in HIV-infected (3.7, 95% CrI: 0.0–10.3) relative to HUU (29.0, 95% CrI: 20.9–39.9). HEU etiology was generally intermediate between HIV-infected and HUU. MTB and Enterobacteriaceae were above 4% in all 3 strata.

## DISCUSSION

We present here updated clinical and etiologic findings among HIV-infected children in Zambia with CAP who are seen in a typical large urban Sub-Saharan African setting, characterized by high HIV prevalence and limited access to quality healthcare. Because PERCH enrollment in Zambia occurred during a period of greater routine childhood vaccine coverage (although limited access to PCV10), nearly universal access to PMTCT regimens, and increased access to pediatric HIV care (including CTM prophylaxis), we believed that our findings would differ from several foundational analyses on causes and outcomes of pneumonia in HIV-infected children conducted from 1990s to 2010.^2,4,7,10,11,41^ However, we found that they are largely similar. Among HIV-infected children hospitalized with severe or very severe pneumonia, common bacterial pathogens, as well as Pj, remained a frequent cause of CXR+ pneumonia; relatively low ART and CTM coverage existed; etiology differed between HIV-infected and uninfected cases and HEU etiology was intermediate; malnutrition was common, and the mortality rate was high.

Six of the top 10 etiologies (*S. pneumoniae*, *H. influenzae*, Enterobacteriaceae, and *S. aureus*, Pj and MTB) are potentially treatable with available antibiotics and antituberculosis medications. While these same 6 organisms were also among the top 10 in our HIV-uninfected analysis,^16^ their cumulative proportion was almost double (75.2% vs. 36.7%) in HIV-infected cases. We recognize the challenges in settings such as UTH in Zambia for conducting routine etiologic and antimicrobial resistance analyses, but periodic analyses, as well as updates to evidence-based treatment guidelines,^42,43^ may be extremely helpful in tailoring local empiric therapy and/or validating current treatment guidelines. The preponderance of bacterial causes (50.6%) may have contributed to the higher in-hospital fatality rate in the HIV-infected cases, compared with HEU (21.6%) and HUU (11.1%) cases at the PERCH Zambia site.

Pj (24.9%) was the most common pathogen attributed as the cause of pneumonia among HIV-infected CXR+ cases (and almost nonexistent among HUU cases at our Zambia site), consistent with prior pneumonia studies and systematic pneumonia etiology reviews among HIV-infected children, and similar to the findings in the South Africa site (22.5%).^2,4,10,11,44^ These findings were also consistent with 2 postmortem studies conducted 15 years apart in Zambia among children dying from reported respiratory causes at UTH.^41,45^ In the Bates study,^45^ conducted at the same time as PERCH, the smaller proportion of histopathologic evidence of Pj (9%) among HIV-infected deaths is likely attributable to an older median age of cases (19 vs. 6 months in PERCH), given the known higher risk during infancy; Pj was nearly absent (0.2%) among PERCH cases over 1 year of age. Due to small sample size, we were unable to determine whether Pj, and lack of CTM prophylaxis, were risk factors for in-hospital death in the multivariate analysis (data not shown). Regardless, Pj continues to play a large etiologic role in HIV-infected pneumonia cases in Zambia, stressing the importance of appropriate CTM prophylaxis and empiric high-dose CTM therapy.

*S. pneumoniae* was the second leading cause for pneumonia among Zambian HIV-infected CXR+ cases (19.8%), consistent with prior pneumonia studies^4,7^ as well as studies reporting increased risk of invasive pneumococcus disease and bacteremia among HIV-infected children.^46,47^ *S. pneumoniae* was much more common among severe versus very severe pneumonia cases [22.8% (95% CrI: 12.2–41.5) vs. 4.3% (95% CrI: 0.0–29.4)]. All serotypes identified by blood culture were covered by PCV10, introduced in Zambia at the end of PERCH enrollment, leading to cautious optimism that PCV10’s introduction may lead to fewer pneumonia cases (and subsequent deaths) in Zambia’s HIV-infected children. Post-PCV10 surveillance in Zambia, particularly for outcomes and etiologic causes of pneumonia in HIV-infected children, will be important.

MTB was estimated to cause 4.5% of CXR+ pneumonia among hospitalized HIV-infected cases in Zambia, 10.1% among HEU (10.1%) and 14.7% among HUU cases. Although these estimates are based on few MTB-positive cases (n = 6), a large systematic review of MTB prevalence among childhood pneumonia cases in similar settings supports MTB as a common cause,^12^ as do 2 Zambian postmortem studies,^41,45^ where MTB was common among acute pneumonia cases. MTB continues to be under-appreciated as a cause of acute pneumonia in children in settings similar to Zambia, and efforts for improving real-time diagnostics, and thus initiating treatment for MTB in children, should be made to improve childhood pneumonia and outcomes.

Despite overall improvements in access to care for HIV-exposed and HIV-infected children such as scaled-up pediatric HIV services, nearly universal access to CTM prophylaxis these beginning at 6 weeks of age, and improved early HIV-1 DNA PCR testing, only 37% of caregivers of HIV-infected cases were aware of their child’s HIV status. Furthermore, both the low number of HIV-infected cases on ART (13.6% overall, 2.6% infants) and on CTM prophylaxis (31.2% of infants) at enrollment indicates an access to healthcare failure. When limiting to HIV-infected children whose caregivers were aware of their status (Supplemental Digital Table 2, http://links.lww.com/INF/D819), these numbers for accessing HIV preventative and definitive care remain surprisingly low, both for cases and controls. Reasons for such limited awareness and access to pediatric HIV care go beyond the scope of this analysis, but this underscores the need for earlier identification of HIV infection status and better caregiver education to ensure that appropriate life-saving chemoprophylaxis and ART can be given.

With 39.8% of cases dying in hospital, outcomes for HIV-infected severe and very severe pneumonia cases at UTH in Lusaka were very poor and much worse than for HEU and HUU children at our site. While similar studies in the region among HIV-infected children show high case fatality rates, notably in Malawi^10^ and South Africa,^7,11^ our in-hospital fatality rate remains alarmingly high. Some likely causes are well-established risk factors for poor outcomes, such as disease severity, younger age and danger signs (Table 2). The larger proportion of bacterial, Pj and MTB causes for pneumonia also likely played a role, particularly with the lack of a standardized approach for care of HIV-infected children with pneumonia among the clinical staff at UTH. Poor immunologic status, high HIV-1 viral load and advanced WHO HIV clinical staging likely influenced outcomes (see below), but these data were unavailable in PERCH. Lack of mechanical ventilation and early resuscitative efforts in an ICU setting likely also contributed.

There were several study limitations particular to the Zambia PERCH site. First, 35% (N = 36) of cases were excluded from the primary etiology analysis due to missing or uninterpretable CXRs. Most missing CXRs were due to staff shortages causing delays in which sicker cases died before obtaining a CXR, or children were deemed too sick to attempt imaging. Second, nearly all children received antibiotics before referral to UTH, likely diminishing bacterial detection by culture and NP/OP PCR; thus, the bacterial fraction is likely underestimated. Third, with poor (~50%) posthospitalization follow-up, we likely underestimated mortality. Fourth, lacking immunologic (CD4 counts and percentages), viral load and HIV clinical staging status, we cannot assess these cofactors’ influence on outcomes and etiology. Fifth, autopsies were not performed, an important though challenging process for informing etiology of fatal cases; however, in the contemporary Bates autopsy study,^45^ evidence of bronchopneumonia (47%), pulmonary mycobacteria (12%) and Pj (9%) were found in HIV-infected children, closely matching our findings.

There are also limitations inherent to pneumonia etiology studies and case–control studies for pneumonia. Although PERCH relied on multiple samples (blood, NP/OP swabs, induced sputum) to estimate etiology, they were taken from locations outside the site of infection (the lung), a common problem for etiology studies. Despite using specimens obtained from outside the lung, some specimen results do have diagnostic potential, either based on standard clinical practice or association with case status, such as NP/OP PCR results for pertussis and RSV or induced sputum for tuberculosis. In addition, for case–control pneumonia etiology studies in children, bacteria which are commonly carried in the nasal passages of children likely lead to an overall underestimation of these bacteria as causative agents. Unfortunately, there is no easy method for distinguishing chronic carriage from newly acquired exposure or infection; however, we have mitigated some of the influence by using quantitative PCR and applied density thresholds that were associated with detection in the blood to improve the value of the data. Finally, the PIA model assumes each case’s pneumonia episode is caused by a single pathogen and it does not attempt to identify or quantify pathogen combinations. While we acknowledge that copathogen causes will result in an underestimate of any single cause, exploratory analyses do not support a large underestimate (data not shown).

These results provide a clinical and microbiologic view of HIV-infected children with severe CAP in a typical Sub-Saharan African setting. These results should be viewed concomitantly and contrasted with the HIV-uninfected results from Zambia^16^ and HIV-infected results from South Africa^44^ in this issue. In aggregate, HIV-infected cases in Zambia had high in-hospital mortality, with common bacterial pathogens, Pj and MTB comprising a large proportion of the etiology. Until improvements in early HIV detection, appropriate CTM prophylaxis and early ART occur, outcomes among HIV-infected children with pneumonia in Zambia will likely remain poor.

## ACKNOWLEDGMENTS

We offer sincere thanks to the children and families who participated in this study. We acknowledge the study of all PERCH contributors who were involved in data collection at the local sites and central laboratories, members of the PERCH Chest Radiograph Reading Panel and Shalika Jayawardena and Rose Watt from Canterbury Health Laboratories. We also acknowledge the substantial contributions of members of the PERCH Study Group:

Johns Hopkins Bloomberg School of Public Health, Baltimore, MD: Orin S. Levine (former principal investigator; current affiliation: Bill & Melinda Gates Foundation, Seattle, WA), Andrea N. DeLuca, Amanda J. Driscoll, Nicholas Fancourt, Wei Fu, E. Wangeci Kagucia, Ruth A. Karron, Mengying Li, Daniel E. Park, Qiyuan Shi, Zhenke Wu, Scott L. Zeger; The Emmes Corporation, Rockville, MD: Nora L. Watson; Nuffield Department of Clinical Medicine, University of Oxford, United Kingdom: Jane Crawley; Medical Research Council, Basse, The Gambia: Stephen R. C. Howie (site principal investigator); KEMRI-Wellcome Trust Research Programme, Kilifi, Kenya: J. Anthony G. Scott (site principal investigator and PERCH co-principal investigator, joint affiliation with London School of Hygiene and Tropical Medicine, London, UK); Division of Infectious Disease and Tropical Pediatrics, Department of Pediatrics, Center for Vaccine Development and Global Health, University of Maryland School of Medicine, Baltimore, MD and Centre pour le Développement des Vaccins (CVD-Mali), Bamako, Mali: Karen L. Kotloff (site principal investigator); Medical Research Council: Respiratory and Meningeal Pathogens Research Unit and Department of Science and Technology/National Research Foundation: Vaccine Preventable Diseases, University of the Witwatersrand, Johannesburg, South Africa: Shabir A. Madhi (site principal investigator); International Centre for Diarrhoeal Disease Research, Bangladesh (icddr,b): W. Abdullah Brooks (site principal investigator); Thailand Ministry of Public Health – U.S. CDC Collaboration, Nonthaburi, Thailand: Henry C. Baggett (site principal investigator), Susan A. Maloney (former site principal investigator); Boston University School of Public Health, Boston, MA, and University Teaching Hospital, Lusaka, Zambia: Donald M. Thea (site principal investigator); Canterbury Health Laboratories, Christchurch, New Zealand: Trevor P. Anderson, Joanne Mitchell.

## Supplementary Material


