# Supplementary material for: The Etiology of Pneumonia in HIV-infected Zambian Children: Findings From the Pneumonia Etiology Research for Child Health (PERCH) Study
Source: Pediatr Infect Dis J. 2021 Aug 25;40(9):S50–8. doi: 10.1097/INF.0000000000002649 (PMC8448411; doi:10.1097/INF.0000000000002649)
Supplement: Supplementary file 2 [file inf-40-s50-s002.docx]

**Supplemental Digital Content 2: HIV characteristics among HIV-infected cases and controls with known HIV-status**

|  | **All Cases** | **CXR+ Cases** | **Controls** |
| --- | --- | --- | --- |
| **All** | 103 | 58 | 85 |
| **HIV characteristics**^a^ |  |  |  |
| **Child reported to be HIV-infected^b^** | 38 (36.9) | 22 (37.9) | 76 (89.4) |
| **Receiving prophylactic cotrimoxazole** | 25 (65.8) | 14 (63.6) | 57 (75.0) |
| **On HAART** | 14 (36.8) | 10 (45.4) | 35 (46.0) |
| **Median weeks on HAART (IQR)^c^** | 17.7 (5.4, 49.3) | 28.4 (5.4, 49.3) | 22.5 (9.6, 58.1) |
| **Attended HAART Clinic in last 3 months** | 19 (50.0) | 13 (59.1) | 40 (52.6) |
| **Had CD4 count measured in last 3 months** | 7 (18.4) | 4 (18.2) | 31 (40.8) |

a. HIV characteristics that were missing or unknown were assumed to be negative.

b. HIV characteristics restricted to those children whose caregivers were aware of their HIV status.

c. Duration restricted to those with available data (N=9/14 cases on HAART and N=34/35 controls on HAART).
