# Supplementary material for: The Etiology of Pneumonia in HIV-infected Zambian Children: Findings From the Pneumonia Etiology Research for Child Health (PERCH) Study
Source: Pediatr Infect Dis J. 2021 Aug 25;40(9):S50–8. doi: 10.1097/INF.0000000000002649 (PMC8448411; doi:10.1097/INF.0000000000002649)
Supplement: Supplementary file 3 [file inf-40-s50-s003.docx]

**Supplemental Digital Content 3. Demographic and clinical characteristics of Cases, by HIV-status**

|  | **All Cases** | | | **CXR+ Cases** | | |
| --- | --- | --- | --- | --- | --- | --- |
|  | **HIV-infected Cases** | **HIV-exposed Cases** | **HIV-unexposed Cases** | **HIV-infected Cases** | **HIV-exposed Cases** | **HIV-unexposed Cases** |
| **All** | 103 | 134 | 369 | 58 | 55 | 151 |
| **Age:** |  |  |  |  |  |  |
| **Median age in months (IQR)** | 6 (3, 12) | 3 (2, 7) | 5 (2, 12) | 6.5 (3, 13) | 3 (1, 6) | 6 (2, 13) |
| **28 days - 5 m** | 51 (49.5) | 89 (66.4) | 185 (50.1) | 27 (46.6) | 39 (70.9) | 71 (47) |
| **6 - 11 m** | 26 (25.2) | 28 (20.9) | 90 (24.4) | 14 (24.1) | 10 (18.2) | 37 (24.5) |
| **12 - 23 m** | 13 (12.6) | 11 (8.2) | 62 (16.8) | 10 (17.2) | 4 (7.3) | 33 (21.9) |
| **24 - 59 m** | 13 (12.6) | 6 (4.5) | 32 (8.7) | 7 (12.1) | 2 (3.6) | 10 (6.6) |
| **Sex:** |  |  |  |  |  |  |
| **Female** | 55 (53.4) | 71 (53) | 159 (43.1) | 29 (50) | 28 (50.9) | 63 (41.7) |
| **Pentavalent (DTP-Hib-HepB) fully vaccinated for age^b^** |  |  |  |  |  |  |
| **< 1 year old** | 54 (73.0) | 82 (70.7) | 200 (77.5) | 29 (74.4) | 32 (66.7) | 79 (78.2) |
| **> 1 year old** | 15 (83.3) | 16 (100) | 73 (92.4) | 8 (72.7) | 6 (100) | 34 (94.4) |
| **Total** | 69 (75.0) | 98 (74.2) | 273 (81.0) | 37 (74.0) | 38 (70.4) | 113 (82.5) |
| **Premature^c^** | 6 (5.8) | 12 (9.0) | 13 (3.5) | 5 (8.6) | 4 (7.3) | 4 (2.7) |
| **HIV characteristics**^d^ |  |  |  |  |  |  |
| **No evidence of prophylactic cotrimoxazole receipt** | 67 (65.1) | 100 (74.6) | 363 (98.4) | 37 (63.8) | 42 (76.4) | 148 (98.0) |
| **Moderate or severe malnutrition (weight-for-age)^e^** | 65 (63.1) | 48 (36.1) | 106 (28.7) | 38 (65.5) | 22 (40.0) | 44 (29.1) |
| **Antibiotic pretreatment prior to specimen collection^f^** | 90 (88.2) | 121 (91.0) | 331 (90.4) | 49 (86.0) | 52 (94.5) | 136 (91.3) |
| **Serum antibiotic activity** | 36 (36.4) | 39 (30.5) | 100 (28.2) | 19 (33.9) | 11 (21.6) | 38 (26.2) |
| **Very severe pneumonia^g^** | 36 (35) | 35 (26.1) | 121 (32.8) | 17 (29.3) | 17 (30.9) | 52 (34.4) |
| **CXR available** | 84 (81.6) | 122 (91.0) | 328 (88.9) | 58 (100) | 55 (100) | 151 (100) |
| **CXR result** |  |  |  |  |  |  |
| **Any consolidation** | 50 (59.5) | 37 (30.3) | 96 (29.3) | 50 (86.2) | 37 (67.3) | 96 (63.6) |
| **Other infiltrate only** | 8 (9.5) | 18 (14.8) | 55 (16.8) | 8 (13.8) | 18 (32.7) | 55 (36.4) |
| **Normal** | 9 (10.7) | 45 (36.9) | 113 (34.5) | 0 (0.0) | 0 (0.0) | 0 (0.0) |
| **Uninterpretable** | 17 (20.2) | 22 (18) | 64 (19.5) | 0 (0.0) | 0 (0.0) | 0 (0.0) |
| **CRP > 40 mg/L** | 46 (48.4) | 42 (35) | 120 (35.8) | 30 (55.6) | 24 (47.1) | 65 (48.1) |
| **Median CRP (mg/L) (IQR)** | 31.0 (5.9, 110.4) | 15.5 (3.0, 62.2) | 23.4 (3.9-62.4) | 56.2 (8.8-171.6) | 36.8 (6.0-73.6) | 34.2 (8.5-91.4) |
| **Severe anemia^h^** | 29 (29.0) | 14 (10.9) | 26 (7.2) | 14 (24.1) | 7 (13.0) | 11 (7.5) |
| **Leukocytosis^i^** | 49 (49.0) | 65 (50.0) | 141 (39.3) | 33 (56.9) | 30 (55.6) | 66 (44.9) |
| **Hypoxia^j^** | 61 (59.8) | 43 (32.1) | 138 (37.5) | 36 (63.2) | 27 (49.1) | 70 (46.4) |
| **Elevated temperature (> 38 C)** | 63 (61.2) | 76 (56.7) | 185 (50.4) | 34 (58.6) | 36 (65.5) | 84 (56.0) |
| **Tachycardia** | 73 (71.6) | 82 (62.1) | 241 (66) | 40 (70.2) | 30 (54.5) | 104 (69.3) |
| **Wheeze on auscultation** | 6 (5.8) | 15 (11.2) | 47 (12.7) | 6 (10.3) | 5 (9.1) | 16 (10.6) |
| **Lethargy^k^** | 17 (16.5) | 12 (9) | 49 (13.3) | 6 (10.3) | 7 (12.7) | 21 (13.9) |
| **Median duration of illness in days (IQR)^l^** | 3 (2, 7) | 3 (2, 5) | 3 (2, 5) | 4 (2, 7) | 3 (2, 7) | 3 (2, 5) |
| **Duration of illness^l^** |  |  |  |  |  |  |
| **0 - 2 days** | 30 (29.1) | 48 (36.4) | 130 (35.3) | 15 (25.9) | 21 (38.2) | 43 (28.5) |
| **3 - 5 days** | 35 (34.0) | 54 (40.9) | 160 (43.5) | 21 (36.2) | 18 (32.7) | 71 (47.0) |
| **>5 days** | 38 (36.9) | 30 (22.7) | 78 (21.2) | 22 (37.9) | 16 (29.1) | 37 (24.5) |
| **Median duration of hospitalization in days (IQR)** | 6 (3, 13) | 5 (2, 8) | 4 (2, 7) | 7 (4, 13) | 5 (2, 8) | 5 (3, 8) |
| **Duration of hospitalization** |  |  |  |  |  |  |
| **0 - 2 days** | 17 (16.7) | 24 (17.9) | 53 (14.4) | 6 (10.5) | 14 (25.5) | 22 (14.7) |
| **3 - 5 days** | 28 (27.5) | 48 (35.8) | 183 (49.7) | 17 (29.8) | 15 (27.3) | 58 (38.7) |
| **>5 days** | 57 (55.9) | 62 (46.3) | 132 (35.9) | 34 (59.6) | 26 (47.3) | 70 (46.7) |
| **Died in hospital** | 41 (39.8) | 29 (21.6) | 41 (11.1) | 16 (27.6) | 11 (20.0) | 12 (7.9) |
| **Died post-discharge, within 30 days of admission** | 2 (3.2) | 1 (1.0) | 5 (1.5) | 1 (5.6) | 1 (2.3) | 1 (0.7) |
| **Missing 30-day vital status^m^** | 31 (50.0) | 43 (41.0) | 170 (51.8) | 24 (57.1) | 17 (38.6) | 63 (45.3) |

Abbreviations: DTP, diptheria-tetanus-pertussis vaccine; m (months); IQR, interquartile range; CXR, chest radiograph; HIV, human immunodeficiency virus.

a. Respiratory tract illness was defined as presence of cough or runny nose, or if a child had (1) at least 1 of ear discharge, wheezing, or difficulty breathing and (2) either a measured temperature of >38.0°C within the previous 48 hours or a history of sore throat.

b. Pentavalent vaccine (DTP-Hib-HepB) used in Zambia. For children <1 year, defined as received at least one dose and up-to-date for age based on the child's age at enrollment, doses received, and country schedule (allowing 4-week window each for dose). For children >1 year, defined as ≥3 doses.

c. Prematurity defined as < 37 weeks gestational age or maternal report of premature.

d. HIV characteristics that were missing were assumed to be negative.

e. Moderate or severe malnutrition defined as < -2 SD weight-for-age z-scores.

f. Defined as serum bioassay positive (cases and controls), antibiotics administered at the referral facility, or antibiotic administration prior to whole-blood specimen collection at the study facility (cases only).

g. Very severe pneumonia defined as cough or difficulty breathing, and at least one of the following: central cyanosis, difficulty breastfeeding/drinking, vomiting everything, convulsions, lethargy, unconsciousness, or head nodding

h. Severe anemia defined as hemoglobin < 7.5 g/dL.

i. Leukocytosis count defined as >15 x 109cells/L for children 1-11 months and >13 x 109 cells/L for children 12-59 months.

j. Hypoxaemia defined as oxygen saturation <90% or on supplemental oxygen if a room air oxygen saturation reading was not available.

k. Lethargic or unresponsive.

l. Duration of illness defined as duration (in days) of cough, wheeze, fever, or difficulty breathing, whichever is longest.

m. Restricted to those children discharged alive.
