# Supplementary material for: The Etiology of Pneumonia in HIV-infected Zambian Children: Findings From the Pneumonia Etiology Research for Child Health (PERCH) Study
Source: Pediatr Infect Dis J. 2021 Aug 25;40(9):S50–8. doi: 10.1097/INF.0000000000002649 (PMC8448411; doi:10.1097/INF.0000000000002649)
Supplement: Supplementary file 4 [file inf-40-s50-s004.docx]

**Supplemental Digital Content 4. Descriptive specimen results by CXR positivity and mortality and specimen type**

| **A. Blood Culture** | |  | |  | |  | |
| --- | --- | --- | --- | --- | --- | --- | --- |
| **ORGANISM** | | **All HIV-Infected N=102** | | **CXR+ HIV-Infected N=57** | | **HIV-Infected Died in Hospital N=41** | |
| **Any^a^** | | 8 (7.8) | | 6 (10.5) | | 3 (7.3) | |
| ***S. pneumoniae*** | | 5 (4.9) | | 4 (7.0) | | 1 (2.4) | |
| ***S. pneumoniae* VT (PCV10)** | | 5 (4.9) | | 4 (7.0) | | 1 (2.4) | |
| ***S. pneumoniae* non-VT (PCV10)** | | 0 (0.0) | | 0 (0.0) | | 0 (0.0) | |
| ***H. influenzae*** | | 1 (1.0) | | 1 (1.8) | | 0 (0.0) | |
| ***H. influenzae* type b** | | 0 (0.0) | | 0 (0.0) | | 0 (0.0) | |
| ***H. influenzae* non-type b** | | 1 (1.0) | | 1 (1.8) | | 0 (0.0) | |
| **Salmonella spp^b^** | | 1 (1.0) | | 0 (0.0) | | 1 (2.4) | |
| ***Klebsiella pneumoniae*^c^** | | 1 (1.0) | | 1 (1.8) | | 1 (2.4) | |
| **B. Induced Sputum Culture** | |  | |  | |  | |
| **ORGANISM** | | **All HIV-Infected N=76** | | **CXR-Infected N=50** | | **HIV-Infected Died in Hospital**  **N=23** | |
| ***Mycobacterium tuberculosis*** | | 1 (1.3) | | 1 (2.0) | | 1 (4.3) | |

a. Excluding contaminants.

b. Other Salmonella species (non-typhoidal).

c. Grouped as ‘Enterobacteriaceae’ for etiology analysis.
