# Supplementary material for: The Etiology of Pneumonia in HIV-infected Zambian Children: Findings From the Pneumonia Etiology Research for Child Health (PERCH) Study
Source: Pediatr Infect Dis J. 2021 Aug 25;40(9):S50–8. doi: 10.1097/INF.0000000000002649 (PMC8448411; doi:10.1097/INF.0000000000002649)
Supplement: Supplementary file 5 [file inf-40-s50-s005.docx]

**Supplemental Table 5. Descriptive specimen results by HIV infection and exposure status, CXR+ Cases**

| **A. Blood Culture** | |  | |  | |  | |
| --- | --- | --- | --- | --- | --- | --- | --- |
| **ORGANISM** | | **CXR+ HIV-Infected N=57** | | **CXR+ HIV-Exposed N=55** | | **CXR+ HIV-Unexposed N=148** | |
| **Any^a^** | | 6 (10.5) | | 3 (5.5) | | 5 (3.4) | |
| ***S. pneumoniae*** | | 4 (7.0) | | 0 (0.0) | | 1 (0.7) | |
| ***S. pneumoniae* VT (PCV10)** | | 4 (7.0) | | 0 (0.0) | | 1 (0.7) | |
| ***S. pneumoniae* non-VT (PCV10)** | | 0 (0.0) | | 0 (0.0) | | 0 (0.0) | |
| ***H. influenzae*** | | 1 (1.8) | | 0 (0.0) | | 1 (0.7) | |
| ***H. influenzae* type b** | | 0 (0.0) | | 0 (0.0) | | 1 (0.7) | |
| ***H. influenzae* non-type b** | | 1 (1.8) | | 0 (0.0) | | 0 (0.0) | |
| ***S. aureus*** | | 0 (0.0) | | 1 (1.8) | | 0 (0.0) | |
| **Salmonella spp^b^** | | 0 (0.0) | | 1 (1.8) | | 2 (1.4) | |
| **Enterobacteriaceae^c^** | | 1 (1.8) | | 1 (1.8) | | 1 (0.7) | |
| ***Candida* species** | | 0 (0.0) | | 0 (0.0) | | 0 (0.0) | |
| **B. Induced Sputum Culture** | |  | |  | |  | |
| **ORGANISM** | | **CXR+ HIV-Infected N=50** | | **CXR+ HIV-Exposed N=47** | | **CXR+ HIV-Unexposed**  **N=133** | |
| **Mycobacterium tuberculosis** | | 1 (2.0) | | 1 (2.1) | | 4 (3.0) | |

a. Excluding contaminants.

b. Includes *S. typhi*, and Other Salmonella spp

c. Enterobacteriaceae includes *Escherichia coli* and *Klebsiella pneumoniae*.
