# Supplementary material for: The Etiology of Pneumonia in HIV-infected Zambian Children: Findings From the Pneumonia Etiology Research for Child Health (PERCH) Study
Source: Pediatr Infect Dis J. 2021 Aug 25;40(9):S50–8. doi: 10.1097/INF.0000000000002649 (PMC8448411; doi:10.1097/INF.0000000000002649)
Supplement: Supplementary file 6 [file inf-40-s50-s006.docx]

**Supplemental Digital Content 6: Codetection of Organisms in NP/OP Specimens Collected from Cases and Controls**

|  | **All Cases N=93** | **CXR+ Cases N=53** | **All Controls N=75** | **All Cases vs All Controls** | **CXR+ Cases vs All Controls** |
| --- | --- | --- | --- | --- | --- |
| Mean (SD) number of organisms, any positivity | 4.38 (1.54) | 4.47 (1.72) | 3.53 (1.47) |  |  |
| Median (IQR) | 4.0 (3.0, 5.0) | 5.0 (3.0, 5.0) | 4.0 (3.0, 4.0) |  |  |
| 0 | 1 (1.1) | 1 (1.9) | 1 (1.3) | 0.1049 | 0.2024 |
| 1 | 2 (2.2) | 1 (1.9) | 6 (8.0) |  |  |
| 2 | 5 (5.4) | 2 (3.8) | 11 (14.7) |  |  |
| 3 | 18 (19.4) | 13 (24.5) | 16 (21.3) |  |  |
| 4 | 67 (72.0) | 36 (67.9) | 41 (54.7) |  |  |
| Mean (SD) number of organisms, above threshold | 3.09 (1.56) | 3.26 (1.64) | 2.00 (1.25) |  |  |
| Median (IQR) | 3.0 (2.0, 4.0) | 3.0 (2.0, 4.0) | 2.0 (1.0, 3.0) |  |  |
| 0 | 4 (4.3) | 2 (3.8) | 8 (10.7) | **0.0260** | **0.0044** |
| 1 | 11 (11.8) | 8 (15.1) | 20 (26.7) |  |  |
| 2 | 20 (21.5) | 5 (9.4) | 24 (32.0) |  |  |
| 3 | 21 (22.6) | 14 (26.4) | 11 (14.7) |  |  |
| 4 | 37 (39.8) | 24 (45.3) | 12 (16.0) |  |  |
| Pathogen patterns, any positivity |  |  |  |  |  |
| Single bacteria | 0 (0.0) | 0 (0.0) | 3 (4.0) | 0.9438 | 0.8714 |
| 2 or more bacteria | 4 (4.3) | 3 (5.7) | 1 (1.3) |  |  |
| Single virus | 2 (2.2) | 1 (1.9) | 3 (4.0) |  |  |
| 2 or more viruses | 3 (3.2) | 2 (3.8) | 2 (2.7) |  |  |
| Bacterial-Viral | 83 (89.2) | 46 (86.8) | 65 (86.7) |  |  |
| Pathogen patterns, above threshold |  |  |  |  |  |
| Single bacteria | 7 (7.5) | 5 (9.4) | 14 (18.7) | 0.2256 | 0.5183 |
| 2 or more bacteria | 5 (5.4) | 3 (5.7) | 6 (8.0) |  |  |
| Single virus | 4 (4.3) | 2 (3.8) | 6 (8.0) |  |  |
| 2 or more viruses | 6 (6.5) | 3 (5.7) | 1 (1.3) |  |  |
| Bacterial-Viral | 66 (71.0) | 37 (69.8) | 40 (53.3) |  |  |

OR adjusted for age in months. For above threshold rows, prevalence defined using NP/OP PCR density thresholds for 4 pathogens: *P. jirovecii*, 4 log_10_ copies/mL; *H. influenzae*, 5.9 log_10_ copies/mL; CMV, 4.9 log_10_ copies/mL; *S. pneumoniae*, 6.9 log_10_ copies/mL.
