# Supplementary material for: The Etiology of Pneumonia in HIV-infected Zambian Children: Findings From the Pneumonia Etiology Research for Child Health (PERCH) Study
Source: Pediatr Infect Dis J. 2021 Aug 25;40(9):S50–8. doi: 10.1097/INF.0000000000002649 (PMC8448411; doi:10.1097/INF.0000000000002649)
Supplement: Supplementary file 7 [file inf-40-s50-s007.docx]

**Supplemental Digital Content 7. Detection of Organisms in Specimens Collected from both HIV-Infected Cases and Controls, by Specimen and Test**

|  | | | | **Odds Ratio (95% CI)** | |
| --- | --- | --- | --- | --- | --- |
|  | **All Cases** | **CXR+ Cases** | **Controls** | **All Cases vs. Controls** | **CXR+ Cases vs. Controls** |
| **NP/OP PCR** | | | | | |
| **Pathogen** | | | | | |
| **Any Pathogen** | 92 (98.9) | 52 (98.1) | 74 (98.7) | 0.41 (0.02, 7.75) | 0.12 (0.01, 2.60) |
| **Any pathogen, with thresholds applied^a^** | 89 (95.7) | 51 (96.2) | 67 (89.3) | 0.68 (0.13, 3.60) | 0.38 (0.04, 3.29) |
| **Bacteria** | | | | | |
| **Any bacteria** | 87 (93.5) | 49 (92.5) | 69 (92.0) | 0.65 (0.10, 4.06) | 0.27 (0.03, 2.54) |
| **Any bacteria, with thresholds applied for *S. pneumoniae, H. influenzae*** | 78 (83.9) | 45 (84.9) | 60 (80.0) | 0.19 (0.03, 1.07) | 0.10 (0.01, 1.10) |
| ***S. pneumoniae*** | 73 (78.5) | 42 (79.2) | 54 (72.0) | 1.39 (0.51, 3.77) | 1.32 (0.37, 4.70) |
| **>6.9 log_10_ copies/ml** | 16 (17.2) | 10 (18.9) | 10 (13.3) | 1.87 (0.59, 5.94) | 3.41 (0.83, 14.08) |
| **Among those with high density *S. pneumoniae* on PCR** |  |  |  |  |  |
| **PCV10-type** | 10 (62.5) | 7 (70.0) | 6 (60.0) | 2.30 (0.55, 9.54) | **7.06 (1.25, 39.84)** |
| **Non PCV10-type** | 6 (37.5) | 4 (40.0) | 3 (30.0) | 1.59 (0.23, 10.98) | 2.15 (0.19, 24.75) |
| ***H. influenzae*** |  |  |  |  |  |
| ***H. influenzae* not type b** | 41 (44.1) | 26 (49.1) | 36 (48.0) | 0.80 (0.35, 1.83) | 1.22 (0.41, 3.62) |
| ***H. influenzae* not type b > 5.9 log_10_ copies/ml** | 20 (21.5) | 15 (28.3) | 9 (12.0) | 2.28 (0.78, 6.59) | **4.80 (1.28, 17.95)** |
| ***H. influenzae* type b** | 7 (7.5) | 3 (5.7) | 5 (6.7) | 0.83 (0.11, 6.05) | 0.04 (0.00, 1.91) |
| ***H. influenzae* type b >5.9 log_10_ copies/ml** | 2 (2.2) | 1 (1.9) | 3 (4.0) | 0.00  (0.00, 29E159) | 0.00 (0.00, 8E262) |
| ***S. aureus*** | 23 (24.7) | 11 (20.8) | 8 (10.7) | **3.13 (1.08, 9.05)** | **4.37 (1.20, 15.85)** |
| ***B. pertussis*** | 1 (1.1) | 0 (0.0) | 0 (0.0) | --^b^ | --^b^ |
| ***C. pneumoniae*** | 0 (0.0) | 0 (0.0) | 0 (0.0) | --^b^ | --^b^ |
| ***Legionella species*** | 0 (0.0) | 0 (0.0) | 0 (0.0) | --^b^ | --^b^ |
| ***M. catarrhalis*** | 70 (75.3) | 43 (81.1) | 52 (69.3) | 0.91 (0.38, 2.19) | 1.32 (0.41, 4.22) |
| ***M. pneumoniae*** | 1 (1.1) | 1 (1.9) | 0 (0.0) | --^b^ | --^b^ |
| **Salmonella species** | 1 (1.1) | 0 (0.0) | 1 (1.3) | 0.51 (0.02, 12.45) | --^b^ |
| **Fungi** | | | | | |
| ***P. jirovecii*** | 29 (31.2) | 16 (30.2) | 5 (6.7) | 5.18 (1.59, 16.87) | 5.35 (1.22, 23.33) |
| ***P. jirovecii* >4 log_10_ copies/ml** | 23 (24.7) | 12 (22.6) | 0 (0.0) | --^b^ | --^b^ |
| **Virus** | | | | | |
| **Any virus** | 88 (94.6) | 49 (92.5) | 70 (93.3) | 1.11 (0.21, 6.00) | 0.42 (0.06, 3.16) |
| **Any virus, with thresholds applied for CMV** | 76 (81.7) | 42 (79.2) | 47 (62.7) | 1.35 (0.32, 5.64) | 0.69 (0.09, 5.19) |
| **Adenovirus** | 10 (10.8) | 5 (9.4) | 2 (2.7) | **10.49 (1.56, 70.72)** | **19.29 (2.13, 174.66)** |
| **CMV** | 83 (89.2) | 47 (88.7) | 64 (85.3) | 1.19 (0.36, 3.92) | 0.51 (0.10, 2.52) |
| **CMV > 4.9 log10 copies/ml** | 52 (55.9) | 32 (60.4) | 27 (36.0) | 0.76 (0.29, 1.99) | 0.64 (0.17, 2.47) |
| **Coronavirus 43** | 5 (5.4) | 2 (3.8) | 2 (2.7) | 4.02 (0.45, 36.20) | 0.80 (0.01, 129.91) |
| **Coronavirus 63** | 3 (3.2) | 1 (1.9) | 2 (2.7) | 2.72 (0.37, 19.98) | 1.85 (0.12, 27.82) |
| **Coronavirus HKU** | 3 (3.2) | 3 (5.7) | 1 (1.3) | 2.66 (0.13, 54.32) | 14.51 (0.53, 400.29) |
| **Coronavirus 229** | 1 (1.1) | 1 (1.9) | 1 (1.3) | 0.22 (0.00, 17.10) | 0.56 (0.00, 118.27) |
| **HBOV** | 11 (11.8) | 8 (15.1) | 11 (14.7) | 0.63 (0.18, 2.19) | 0.32 (0.06, 1.66) |
| **HMPV A/B** | 4 (4.3) | 1 (1.9) | 2 (2.7) | 0.90 (0.09, 9.40) | 0.54 (0.02, 17.45) |
| **Influenza A** | 2 (2.2) | 0 (0.0) | 0 (0.0) | --^b^ | --^b^ |
| **Influenza B** | 1 (1.1) | 0 (0.0) | 1 (1.3) | 1.38 (0.07, 28.28) | --^b^ |
| **Influenza C** | 1 (1.1) | 1 (1.9) | 0 (0.0) | --^b^ | --^b^ |
| **Parainfluenza 1** | 1 (1.1) | 1 (1.9) | 0 (0.0) | --^b^ | --^b^ |
| **Parainfluenza 2** | 0 (0.0) | 0 (0.0) | 0 (0.0) | --^b^ | --^b^ |
| **Parainfluenza 3** | 2 (2.2) | 2 (3.8) | 1 (1.3) | 2.74 (0.12, 64.69) | 4.49 (0.17, 117.93) |
| **Parainfluenza 4** | 4 (4.3) | 3 (5.7) | 0 (0.0) | --^b^ | --^b^ |
| **PV/EV** | 4 (4.3) | 3 (5.7) | 2 (2.7) | 0.81 (0.05, 13.83) | 1.63 (0.07, 37.82) |
| **Rhinovirus** | 19 (20.4) | 10 (18.9) | 13 (17.3) | 1.18 (0.40, 3.4772) | 0.84 (0.19, 3.68) |
| **RSV** | 7 (7.5) | 7 (13.2) | 2 (2.7) | 4.14 (0.69, 24.73) | **9.81 (1.34, 72.02)** |
| **Whole Blood PCR** | | | | | |
| ***S. pneumoniae*** | 13 (14.4) | 8 (13.8) | 7 (10.0) | 1.61 (0.58, 4.45) | 1.68 (0.55, 5.15) |
| **>2.2 log_10_ copies/ml** | 9 (10.0) | 6 (10.3) | 3 (4.3) | 2.76 (0.68, 11.20) | 2.98 (0.67, 13.20) |

NP/OP OR adjusted for age in months and presence of other pathogens. Whole blood OR adjusted for age in months.

1. Threshold defined using NP/OP PCR density for 4 pathogens: *P. jirovecii*, 4 log10 copies/mL; *H. influenzae*, 5.9 log10 copies/mL; CMV, 4.9 log10 copies/mL; *S. pneumoniae*, 6.9 log10 copies/mL).

b. Odd ratios could not be calculated due to zero cells.
