# Supplementary material for: The Etiology of Pneumonia in HIV-infected Zambian Children: Findings From the Pneumonia Etiology Research for Child Health (PERCH) Study
Source: Pediatr Infect Dis J. 2021 Aug 25;40(9):S50–8. doi: 10.1097/INF.0000000000002649 (PMC8448411; doi:10.1097/INF.0000000000002649)
Supplement: Supplementary file 8 [file inf-40-s50-s008.docx]

**Supplemental Digital Content 8. Detection of Organisms in Specimens Collected from both Cases and Controls, by HIV-status, Specimen and Test**

|  | **HIV-infected** | | | **HIV-exposed** | | | | **HIV-unexposed** | | |
| --- | --- | --- | --- | --- | --- | --- | --- | --- | --- | --- |
|  | **CXR+ Cases (N=53)** | **Controls (N=75)** | **Odds Ratio (95% CI)** | **CXR+ Cases (N=52)** | **Controls (N=150)** | **Odds Ratio (95% CI)** | **CXR+ Cases (N=139)** | | **Controls (N=373)** | **Odds Ratio (95% CI)** |
| **NP/OP PCR** |  |  |  |  |  |  |  | |  |  |
| **Bacteria** | | | | | | | | | | |
| **Any bacteria** | 49 (92.5) | 69 (92.0) | 0.27 (0.03, 2.54) | 48 (92.3) | 137 (91.3) | 1.24 (0.24, 6.43) | 130 (93.5) | | 354 (94.9) | 0.51 (0.17, 1.47) |
| **Any bacteria, with thresholds applied for *S. pneumoniae, H. influenzae*** | 45 (84.9) | 60 (80.0) | 0.10 (0.01, 1.10) | 38 (73.1) | 119 (79.3) | 0.51 (0.05, 4.73) | 115 (82.7) | | 328 (87.9) | 0.45 (0.14, 1.45) |
| ***S. pneumoniae*** | 42 (79.2) | 54 (72.0) | 1.32 (0.37, 4.70) | 37 (71.2) | 116 (77.3) | 0.46 (0.17, 1.26) | 111 (79.9) | | 307 (82.3) | 0.99 (0.51, 1.91) |
| **>6.9 log_10_ copies/ml** | 10 (18.9) | 10 (13.3) | 3.41 (0.83, 14.08) | 5 (9.6) | 9 (6.0) | 2.72 (0.49, 14.99) | 10 (7.2) | | 20 (5.4) | 1.04 (0.39, 2.78) |
| **Among those with high density *S. pneumoniae* on PCR** |  |  |  |  |  |  |  | |  |  |
| **PCV10-type** | 7 (70.0) | 6 (60.0) | 7.06 (1.25, 39.84) | 0 (0.0) | 6 (66.7) | --^b^ | 6 (60.0) | | 10 (50.0) | 1.36 (0.38, 4.85) |
| **Non PCV10-type** | 4 (40.0) | 3 (30.0) | 2.15 (0.19, 24.75) | 4 (80.0) | 2 (22.2) | 17.51 (1.67, 183.90) | 4 (40.0) | | 9 (45.0) | 0.80 (0.18, 3.59) |
| ***H. influenzae*** |  |  |  |  |  |  |  | |  |  |
| ***H. influenzae* not type b** | 26 (49.1) | 36 (48.0) | 1.09 (0.35, 3.33) | 28 (53.8) | 45 (30.0) | 3.03 (1.27, 7.23) | 61 (43.9) | | 151 (40.5) | 1.10 (0.66, 1.82) |
| ***H. influenzae* not type b > 5.9 log_10_ copies/ml** | 15 (28.3) | 9 (12.0) | 4.39 (1.18, 16.30) | 10 (19.2) | 18 (12.0) | 1.09 (0.31, 3.90) | 27 (19.4) | | 42 (11.3) | 1.54 (0.79, 3.00) |
| ***H. influenzae* type b** | 3 (5.7) | 5 (6.7) | 0.08 (0.00, 3.64) | 1 (1.9) | 4 (2.7) | 0.75 (0.04, 13.52) | 9 (6.5) | | 13 (3.5) | 3.04 (1.06, 8.74) |
| ***H. influenzae* type b >5.9 log_10_ copies/ml** | 1 (1.9) | 3 (4.0) | 0.00 (0.00, 8E262) | 0 (0) | 3 (2.0) | --^b^ | 5 (3.6) | | 4 (1.1) | 5.71 (1.11, 29.47) |
| ***S. aureus*** | 11 (20.8) | 8 (10.7) | 4.20 (1.17, 15.11) | 10 (19.2) | 12 (8.0) | 2.39 (0.72, 7.99) | 22 (15.8) | | 50 (13.4) | 0.96 (0.48, 1.92) |
| ***B. pertussis*** | 0 (0.0) | 0 (0.0) | --^b^ | 0 (0.0) | 0 (0.0) | --^b^ | 3 (2.2) | | 0 (0.0) | --^b^ |
| ***C. pneumoniae*** | 0 (0.0) | 0 (0.0) | --^b^ | 0 (0.0) | 0 (0.0) | --^b^ | 0 (0.0) | | 3 (0.8) | --^b^ |
| ***M. catarrhalis*** | 43 (81.1) | 52 (69.3) | 1.25 (0.38, 4.05) | 34 (65.4) | 112 (74.7) | 0.61 (0.25, 1.51) | 97 (69.8) | | 300 (80.4) | 0.55 (0.31, 0.98) |
| ***M. pneumoniae*** | 1 (1.9) | 0 (0.0) | 526E8 (0.00, I) | 0 (0.0) | 0 (0.0) | -- | 0 (0.0) | | 0 (0.0) | -- |
| **Salmonella species** | 0 (0.0) | 1 (1.3) | --^b^ | 1 (1.9) | 0 (0.0) | --^b^ | 1 (0.7) | | 0 (0.0) | --^b^ |
| **Fungi** |  |  |  |  |  |  |  | |  |  |
| ***P. jirovecii*** | 16 (30.2) | 5 (6.7) | 5.28 (1.21, 22.98) | 11 (21.2) | 11 (7.3) | 5.73 (1.74, 18.81) | 14 (10.1) | | 45 (12.1) | 0.76 (0.35, 1.67) |
| **>4 log_10_ copies/ml** | 12 (22.6) | 0 (0.0) | --^b^ | 6 (11.5) | 2 (1.3) | 15.99 (2.46, 103.80) | 6 (4.3) | | 7 (1.9) | 2.49 (0.71, 8.69) |
| **Virus** | | | | | | | | | | |
| **Any virus** | 49 (92.5) | 70 (93.3) | 0.42 (0.06, 3.16) | 45 (86.5) | 118 (78.7) | 1.43 (0.38, 5.33) | 118 (84.9) | | 279 (74.8) | 1.14 (0.55, 2.36) |
| **Any virus, with thresholds applied for CMV** | 42 (79.2) | 47 (62.7) | 0.69 (0.09, 5.19) | 39 (75.0) | 91 (60.7) | 1.62 (0.40, 6.53) | 109 (78.4) | | 209 (56.0) | 1.87 (0.82, 4.25) |
| **Adenovirus** | 5 (9.4) | 2 (2.7) | 20.30 (2.16, 190.52) | 3 (5.8) | 6 (4.0) | 4.48 (0.86, 23.31) | 11 (7.9) | | 25 (6.7) | 1.41 (0.58, 3.43) |
| **CMV** | 47 (88.7) | 64 (85.3) | 0.51 (0.10, 2.51) | 30 (57.7) | 100 (66.7) | 0.74 (0.31, 1.73) | 69 (49.6) | | 205 (55.0) | 0.92 (0.57, 1.49) |
| **> 4.9 log10 copies/ml** | 32 (60.4) | 27 (36.0) | 0.66 (0.17, 2.54) | 16 (30.8) | 38 (25.3) | 0.75 (0.28, 2.01) | 29 (20.9) | | 69 (18.5) | 0.99 (0.54, 1.81) |
| **Coronavirus 43** | 2 (3.8) | 2 (2.7) | 0.82 (0.01, 131.11) | 3 (5.8) | 2 (1.3) | 11.55 (0.79, 169.18) | 1 (0.7) | | 9 (2.4) | 0.33 (0.03, 3.70) |
| **Coronavirus 63** | 1 (1.9) | 2 (2.7) | 1.90 (0.13, 28.09) | 1 (1.9) | 7 (4.7) | 0.50 (0.05, 5.14) | 2 (1.4) | | 16 (4.3) | 0.35 (0.06, 1.92) |
| **Coronavirus HKU** | 3 (5.7) | 1 (1.3) | 14.49 (0.50, 416.10) | 1 (1.9) | 5 (3.3) | 0.45 (0.03, 7.10) | 3 (2.2) | | 12 (3.2) | 0.50 (0.08, 3.25) |
| **Coronavirus 229** | 1 (1.9) | 1 (1.3) | 0.49 (0.00, 105.61) | 1 (1.9) | 1 (0.7) | 1.56 (0.02, 159.88) | 2 (1.4) | | 2 (0.5) | 2.65 (0.19, 36.32) |
| **HBOV** | 1 (1.9) | 2 (2.7) | 0.54 (0.02, 17.58) | 7 (13.5) | 11 (7.3) | 1.73 (0.44, 6.82) | 20 (14.4) | | 40 (10.7) | 1.69 (0.83, 3.42) |
| **HMPV A/B** | 0 (0.0) | 0 (0.0) | --^b^ | 5 (9.6) | 3 (2.0) | 12.94 (2.62, 63.97) | 20 (14.4) | | 7 (1.9) | 15.37 (5.77, 40.95) |
| **Influenza A** | 0 (0) | 1 (1.3) | --^b^ | 3 (5.8) | 3 (2.0) | 4.14 (0.59, 28.86) | 8 (5.8) | | 4 (1.1) | 6.79 (1.55, 29.66) |
| **Influenza B** | 1 (1.9) | 0 (0.0) | --^b^ | 0 (0.0) | 0 (0.0) | --^b^ | 1 (0.7) | | 0 (0.0) | --^b^ |
| **Influenza C** | 1 (1.9) | 0 (0.0) | --^b^ | 0 (0.0) | 1 (0.7) | --^b^ | 0 (0) | | 2 (0.5) | --^b^ |
| **Parainfluenza 1** | 0 (0.0) | 0 (0.0) | --^b^ | 0 (0.0) | 1 (0.7) | --^b^ | 1 (0.7) | | 0 (0.0) | --^b^ |
| **Parainfluenza 2** | 2 (3.8) | 1 (1.3) | 4.31 (0.16, 113.03) | 0 (0.0) | 0 (0.0) | --^b^ | 0 (0.0) | | 0 (0.0) | --^b^ |
| **Parainfluenza 3** | 3 (5.7) | 0 (0) | --^b^ | 4 (7.7) | 3 (2.0) | 7.50 (1.03, 54.43) | 7 (5.0) | | 11 (2.9) | 2.66 (0.88, 8.02) |
| **Parainfluenza 4** | 3 (5.7) | 2 (2.7) | 1.71 (0.07, 40.27) | 2 (3.8) | 2 (1.3) | 3.95 (0.22, 71.86) | 3 (2.2) | | 5 (1.3) | 2.10 (0.42, 10.43) |
| **PV/EV** | 10 (18.9) | 13 (17.3) | 0.94 (0.21, 4.17) | 0 (0.0) | 13 (8.7) | --^b^ | 6 (4.3) | | 21 (5.6) | 0.50 (0.15, 1.70) |
| **Rhinovirus** | 7 (13.2) | 2 (2.7) | 10.15 (1.37, 75.11) | 10 (19.2) | 30 (20.0) | 1.80 (0.68, 4.81) | 22 (15.8) | | 62 (16.6) | 1.43 (0.75, 2.71) |
| **RSV** | 1 (1.9) | 2 (2.7) | 0.54 (0.02, 17.58) | 8 (15.4) | 7 (4.7) | 4.35 (1.20, 15.81) | 32 (23.0) | | 11 (2.9) | 14.28 (6.55, 31.12) |
| **Whole Blood PCR** | | | | | | | | | | |
| *S. pneumoniae* | 8 (14.8) | 7 (10.0) | 1.68 (0.55, 5.15) | 6 (12.0) | 9 (5.7) | 2.31 (0.77, 6.94) | 12 (9.2) | | 15 (4.1) | 2.40 (1.09, 5.27) |
| > 2.2 log10 copies/ml | 6 (11.1) | 3 (4.3) | 2.98 (0.67, 13.2) | 5 (10.0) | 5 (3.2) | 3.46 (0.94, 12.75) | 6 (4.6) | | 9 (2.4) | 1.96 (0.68, 5.62) |

NP/OP OR adjusted for age in months and presence of other pathogens. Whole blood OR adjusted for age in months.

a. Threshold defined using NP/OP PCR density for 4 pathogens: *P. jirovecii*, 4 log10 copies/mL; *H. influenzae*, 5.9 log10 copies/mL; CMV, 4.9 log10 copies/mL; *S. pneumoniae*, 6.9 log10 copies/mL).

b. Odd ratios could not be calculated due to zero cells.
