# Supplementary material for: The Etiology of Pneumonia in HIV-infected Zambian Children: Findings From the Pneumonia Etiology Research for Child Health (PERCH) Study
Source: Pediatr Infect Dis J. 2021 Aug 25;40(9):S50–8. doi: 10.1097/INF.0000000000002649 (PMC8448411; doi:10.1097/INF.0000000000002649)
Supplement: Supplementary file 9 [file inf-40-s50-s009.docx]

**Supplemental Digital Content 9. Etiologic fraction for top ten pathogens among HIV+ cases**

|  | **Etiologic Fraction (95% CI)** |
| --- | --- |
| **Aetiology** | **All CXR+**  **(n=58)** |
| **Bacteria** |  |
| *B. pertussis* | 0.2 (0.0, 1.7) |
| *C. pneumoniae* | 0.2 (0.0, 1.7) |
| Enterobacteriaceae | 6.4 (1.7, 19.0) |
| *H. influenzae* | 6.8 (1.7, 17.2) |
| Type b | 1 (0, 6.9) |
| Non-b | 5.8 (1.7, 15.5) |
| Legionella species | 0.7 (0.0, 6.9) |
| *M. catarrhalis* | 0.9 (0.0, 6.9) |
| *M. pneumoniae* | 0.5 (0.0, 3.4) |
| *M. tuberculosis* | 4.5 (1.7, 12.1) |
| *N. meningitides* | 0.8 (0.0, 6.9) |
| Non-fermenting gram-negative rods | 1.0 (0.0, 8.6) |
| Other streptococci and enterococci | 1.0 (0.0, 8.6) |
| *S. aureus* | 12.7 (0.0, 25.9) |
| *S. pneumoniae* | 19.8 (8.6, 36.2) |
| VT | 14.6 (5.2, 29.3) |
| NVT | 5.2 (1.7, 13.8) |
| Salmonella species | 0.4 (0.0, 3.4) |
| **Fungi** |  |
| *Candida* species | 0.9 (0.0, 8.6) |
| *P. jirovecii* | 24.9 (15.5, 36.2) |
| **Viruses** |  |
| Adenovirus | 4.6 (0.0, 13.8) |
| Bocavirus | 0.7 (0.0, 5.2) |
| CMV | 1.0 (0.0, 10.3) |
| Coronavirus | 0.6 (0.0, 5.2) |
| HMPV A/B | 0.4 (0.0, 3.4) |
| Influenza | 0.8 (0.0, 5.2) |
| A | 0.2 (0.0, 1.7) |
| B | 0.2 (0.0, 1.7) |
| C | 0.5 (0.0, 3.4) |
| Parainfluenza | 2.6 (0.0, 10.3) |
| 1 | 0.9 (0.0, 5.2) |
| 2 | 0.2 (0.0, 1.7) |
| 3 | 0.6 (0.0, 5.2) |
| 4 | 1 (0.0, 6.9) |
| PV/EV | 2.0 (0.0, 8.6) |
| Rhinovirus | 0.8 (0.0, 5.2) |
| RSV A/B | 3.7 (0.0, 10.3) |
| Not otherwise specified | 1.2 (0.0, 8.6) |
| **Summary Estimates** |  |
| Bacteria^a^ | 50.6 (32.8, 67.2) |
| Viruses | 17.1 (5.2, 31.0) |

Red indicates pathogens in the Top 10 among CXR+/HIV+ cases.

a. Bacterial summary estimate excludes *M. tuberculosis*.
